# Toxic effects of the neem oil (*Azadirachta indica*) formulation on the stink bug predator, *Podisus nigrispinus* (Heteroptera: Pentatomidae)

**DOI:** 10.1038/srep30261

**Published:** 2016-09-06

**Authors:** José Cola Zanuncio, Sheila Abreu Mourão, Luis Carlos Martínez, Carlos Frederico Wilcken, Francisco S. Ramalho, Angelica Plata-Rueda, Marcus Alvarenga Soares, José Eduardo Serrão

**Affiliations:** 1 Departamento de Entomologia, BIOAGRO, Universidade Federal de Viçosa, 36570-900, Viçosa, Minas Gerais, Brasil; 2 Departamento de Fitotecnia, Universidade Federal de Viçosa, 36570-900, Viçosa, Minas Gerais, Brasil; 3 Departamento de Biologia Geral, Universidade Federal de Viçosa, 36570-900, Viçosa, Minas Gerais, Brasil; 4 Departamento de Proteção de Plantas, Escola de Ciências Agronômicas, Universidade Estadual Paulista, 18603-970, Botucatu, Brasil; 5 Unidade de Controle Biológico, Embrapa Algodão, Av. Osvaldo Cruz, 1143, Campina Grande-PB, CEP 58428-095, Brasil; 6 Departamento de Agronomia, Universidade Federal dos Vales do Jequitinhonha e Mucuri, 391000-000 Diamantina, Minas Gerais, Brasil

## Abstract

This research investigated the effects of neem oil on mortality, survival and malformations of the non-target stink bug predator, *Podisus nigrispinus*. Neurotoxic and growth inhibitor insecticides were used to compare the lethal and sublethal effects from neem oil on this predator. Six concentrations of neem oil were topically applied onto nymphs and adults of this predator. The mortality rates of third, fourth, and fifth instar nymphs increased with increasing neem oil concentrations, suggesting low toxicity to *P. nigrispinus* nymphs. Mortality of adults was low, but with sublethal effects of neem products on this predator. The developmental rate of *P. nigrispinus* decreased with increasing neem oil concentrations. Longevity of fourth instar nymphs varied from 3.74 to 3.05 d, fifth instar from 5.94 to 4.07 d and adult from 16.5 and 15.7 d with 0.5 and 50% neem doses. *Podisus nigrispinus* presented malformations and increase with neem oil concentrations. The main malformations occur in wings, scutellum and legs of this predator. The neem oil at high and sub lethal doses cause mortality, inhibits growth and survival and results in anomalies on wings and legs of the non-traget predator *P. nigrispinus* indicating that its use associated with biological control should be carefully evaluated.

Botanical insecticides have eco-toxicological advantages compared to traditional synthetic insecticides, because they can have favorable eco-toxicological properties (low human toxicity, rapid degradation and reduced environmental impact), which make them suitable insecticides for organic agriculture^1^,^2^,^3^. Botanical insecticides have secondary metabolites such as alkaloids, amides, chalcones, flavones, phenols, lignans, neolignans or kawapirones; which are important in plant-insect interactions and may be used in integrated pest management (IPM) programs^2^,^4^,^5^,^6^. They act as repellents with unpleasant odors or irritants, growth regulators and have deterrence on oviposition and feeding, and biocide activity^1^,^3^,^6^.

The neem oil, *Azadirachta indica* A. Juss (Sapindales: Meliaceae) have insecticide effect against pests as reported for Coleoptera^7^,^8^, Diptera^9^,^10^, Hemiptera^11^,^12^,^13^, and Lepidoptera^14^,^15^.

Azadirachtin is the main compound of the neem oil with insecticidal activity and can be found in fruits and leaves^16^,^17^,^18^. Other neem oil compounds (tetranortriterpenoids group) are desacetylnimbin, desacetylsalannin, nimbin and salannin^18^.

The neem oil is a feeding inhibitor, delaying development and growth, reducing fecundity and fertility, changing behavior and causing anomalies in eggs, larvae and adults of insects or mites^7^,^19^,^20^,^21^.

Azadirachtin has higher toxicity by ingestion than by contact, which allows it to be used somewhat selectively against phytophagous pests^22^,^23^. Although natural enemies may ingest contaminated prey, adverse indirect effects may be negligible because 90% of azadirachtin consumed is eliminated from the body of phytophagous insects seven hours after ingestion^24^.

The stink bug predator, *Podisus nigrispinus* Dallas (Heteroptera: Pentatomidae) is a common zoophytophagous insect that is used in the biological control of agriculture and forest pests in the America^25^,^26^,^27^,^28^. The potential of *P. nigrispinus* in biological control has been reported for larvae of the defoliators *Alabama argillacea* Hübner, *Anticarsia gemmatalis* Hübner, *Spodoptera exigua* Hübner, and *Trichoplusia ni* Hübner (Lepidoptera: Noctuidae)^28^,^29^,^30^,^31^,^32^ in cotton, tomato, soybean, and *Eucalyptus* plantations^27^,^33^,^34^,^35^. The compatibility between *P. nigrispinus* and pesticides such as chlorantraniliprole, deltamethrin, imidacloprid, methamidophos, spinosad and thiamethoxam has been successfully demonstrated^36^,^37^.

Despite the potential benefits of neem oil, this product is mainly sprayed directly onto plant leaves^12^,^38^, which exposes non-target species to this compound^36^,^39^. Although these studies shows that neem oil can be used in IPM programs, they are limited for a few number of species, additional studies of the potential of direct and indirect effects of neem oil on other natural enemies are yet necessary before it can be recommended for IPM programs.

The objective of this study was to evaluate the toxic effects of topical applications of different concentrations of neem oil on the survival and development of the non-target predator *P. nigrispinus*.

## Results

### Comparative toxicity of neem oil and insecticides against *P. nigrispinus*

The highest mortalities of this predator were obtained with 50 and 100 μL mL^−1^ of neem oil, pyriproxifen, and imidacloprid. The two different lethal concentrations levels (LC_50_ and LC_90_) (Table 1) of each treatment was estimated by Probit (*X*^*2*^; *P* < 0.001). The LC_50_ and LC_90_ values indicated that pyriproxifen (*X*^*2*^ = 28.55; df = 5) and imidacloprid (*X*^*2*^ = 23.67; df = 5) were the most toxic compounds to the *P. nigrispinus* nymphs followed by neem oil (*X*^*2*^ = 14.29; df = 5). The lethal concentrations (LC_50-90_) value of the pyriproxifen (*X*^*2*^ = 28.51; df = 5), neem oil (*X*^*2*^ = 27.96; df = 5), and imidacloprid (*X*^*2*^ = 25.64; df = 5) showed the toxicicity of these compounds to the *P. nigrispinus* adults. Mortality was always <1% in the control.

### Lethal effect of neem oil on *P. nigrispinus*

**Figure 1:**
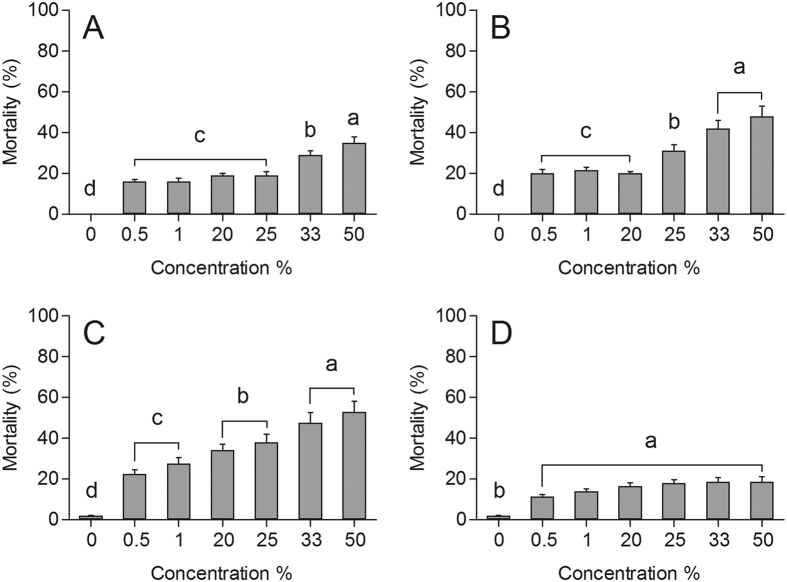
Mortality of *Podisus nigrispinus* (Heteroptera: Pentatomidae) nymphs after topical application of the neem oil: third (**A**) fourth (**B**) and fifth (**C**) instar and adults (**D**). Concentrations means (percent mortality ± SEM) differ significantly at *P* < 0.05 (Tukey’s mean separation test).

The neem oil had lethal effect on the third, fourth and fifth instar nymphs and adults *P. nigrispinus* (Fig. 1). Mortality rates of third instar *P. nigrispinus* nymphs were directly proportional to the neem oil concentrations with values of 15.38, 15.38, 19.23, 19.23, 28.46 and 34.61% (F_1,51_ = 3.24; P < 0.05) with the concentrations of 0.5, 1, 20, 25, 33 and 50%, respectively (Fig. 1A). The mortality increased in fourth instar to 23.07, 26.92, 34.61, 38.46, 46.15 and 53.84% with increasing neem oil concentrations (F_1,51_ = 6.56; P < 0.05) (Fig. 1B). The mortality caused on fifth instar differed with the neem oil concentrations (F_1,51_ = 11.93; P < 0.05) (Fig. 1C). Mortality of *P. nigrispinus* adults was proportional to the neem oil concentration with values of 11.49, 13.91, 16.66, 17.98, 18.51, and 18.66% (F_1,51_ = 3.12; P < 0.05), in the concentrations of 0.5, 1, 20, 25, 33 and 50%, respectively (Fig. 1A). Mortality never exceeded 2% in the control.

### Development and survival

The developmental rate of *P. nigrispinus* varied at different concentrations for fourth (F_1,137_ = 2.19; P < 0.05) and fifth (F_1,137_ = 5.10; P < 0.05) instars and adults (F_1,137_ = 1.45; P < 0.05) compared to the control (Table 2). Nymph longevity decreased with increasing concentrations of neem oil (0.5 > 1 > 20 > 25 > 33 > 50%). Fourth instar longevity varied from 3.74 to 3.05 d, the fifth from 5.94 to 4.07 d and that of adult between 16.5 and 15.7 d from 0.5 to 50% of neem oil.

Neem oil concentrations had a strong effect on the survival of nymph to adult *P. nigrispinus* from the fourth instar (Table 3). This survival varied with concentrations of this oil for fourth (F_1,137_ = 5.24; P < 0.05) and fifth (F_1,137_ = 7.53; P < 0.05) instar nymphs and adults (F_1,137_ = 3.47; P < 0.05). In general, survival rates declined at 20, 25, 33 and 50% neem oil concentrations.

### Malformations caused for the neem oil

Nymphs and adults *P. nigrispinus* presented malformations (*X*^*2*^, P < 0.0001) with its number increasing proportionally with the neem concentrations (0.5 > 1 > 20 > 25 > 33 > 50%) (Table 3). Malformations in the fifth instar nymphs varied from 1.9 to 21.6% with 0.5 to 50% concentrations of neem oil, whereas it ranged from 2.5 to 30.8% in adults.

**Figure 2:**
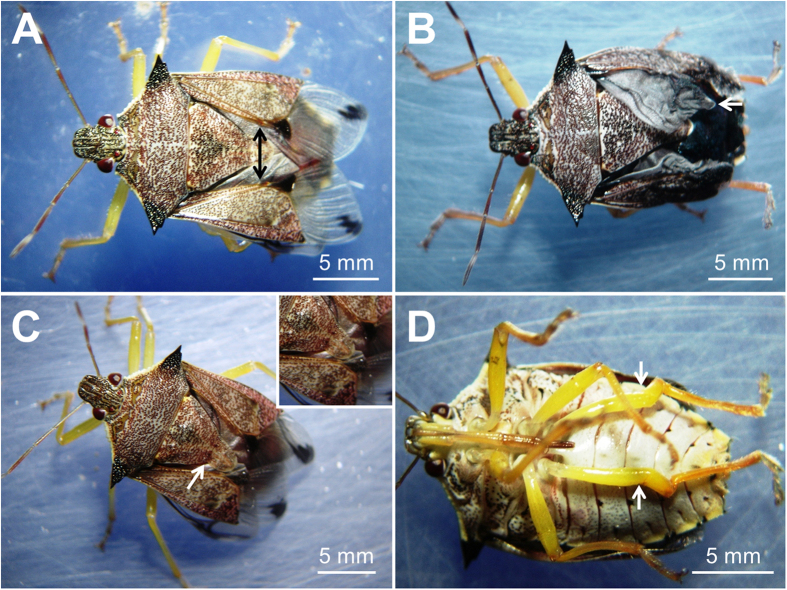
Malformations in adults *Podisus nigrispinus* (Heteroptera: Pentatomidae) after topical application of neem oil on its nymphs. (**A**) Defective hemelytra (arrow), (**B**) low number of veins and membranous area of wings (arrow), (**C**) asymmetric scutellum (arrow) and, (**D**) extension and folding the legs (arrows).

Irreversible malformations occurred in *P. nigrispinus* adults with higher severity as the neem concentrations increased. The main malformations were the hemelytra size reduction (Fig. 2A), low number of veins and reduced membranous area of wings (Fig. 2B), asymmetric scutellum (Fig. 2C), and extension and folding of the legs (Fig. 2D).

## Discussion

Chemical and biological pest control procedures need high selectivity for use in IPM programs, for instance, broad-spectrum insecticides are not suitable. Selectivity of insecticides can be achieved with environmental measures, by minimizing exposure to predators, or physiologically, with insecticides that are more toxic to pests than to predators^6^,^11^,^40^.

The toxicity profiles for the neem oil compared to two insecticides as positive control on the stink bug predator, *P. nigrispinus* were determined from the bioassays. The neem oil, pyriproxifen, and imidacloprid caused substantial mortality of *P. nigrispinus* nymphs and adults under laboratory conditions. The susceptibility of the Hemiptera may vary with exposure in the different concentrations of neem oil and insecticides^41^,^42^,^43^. The LC_50_ and LC_90_ values indicated that lethality of neem oil and imidacloprid were lower on *P. nigrispinus* than pyriproxifen with the concentrations evaluated. However, the lethality of neem oil is confirmed on *N. nigrispinus* depending on the concentration applied and can be compared to neurotoxic insecticides and growth inhibitors, as a potent natural insecticide. Mortality of *P. nigrispinus* was not restricted to the third instar nymphs. Lethal effect on adult stage is also apparent even when exposed during the first hours. This is a common toxic effect of pyriproxyfen and imidacloprid, and also was observed with the neem oil. As would be expected for a juvenile hormone (JH) mimic as the neem oil and pyriproxyfen, which also leads to adult malformation and reproductive impairment of individuals emerged^41^,^43^.

Here, the term selectivity refers to the toxicological selectivity of neem oil on non-target organism as *P. nigrispinus*. Our results showed that neem oil at different concentrations (i.e., 0.5, 1, 20, 25, 33 and 50%) affected the nymphs and adults *P. nigrispinus.* Survival was higher in adults (91.4%) than in fourth (72.3%) and fifth (76.4%) instar nymphs, indicating that *P nigrispinus* was more susceptible in the immature stages. Previous studies on its prey, *Anticarsia gemmatalis* Hübner (Lepidoptera: Noctuidae), show 18.5% larval survival at 25% concentration of neem oil and displayed abnormalities after the last larval moult^44^. Sub-lethal effects on this insect may greatly hinder the survival, and fitness of nymphs with malformations in the adults of this predator and its prey.

The concentrations of neem oil caused mortality on the third, fourth, and fifth instar nymphs. On the other hand, neem as a botanical pesticide has many excellent attributes including its broad-spectrum in insect growth regulatory effects, systemic action in some plants, minimal effects on natural enemies and pollinators, rapid degradation in the environment, and no toxicity to vertebrates^2^,^45^,^46^. The increased mortality of third, fourth and fifth instar *P. nigrispinus* nymphs may be a sublethal effects of the neem oil on this predator. *Spodoptera littoralis* Boisduval (Lepidoptera: Noctuidae) larvae died before the pupa stage after application of 0.5 ppm azadirachtin, probably due to feeding inhibition^47^. The stink bug predator, *Macrolophus caliginosus* Wagner (Hemiptera: Miridae) showed similar chronic toxicity in all instars with different neem oils^11^. Oil from neem seeds containing 0.05 and 0.1 g/L of azadirachtin reduces population growth of the pest aphid *Myzus persicae* Sulzer (Hemiptera: Aphididae) and had sublethal effects on the predatory ladybird beetle *Eriopis connexa* Germar (Coleoptera: Coccinellidae)^8^.

The longevity of fourth and fifth instar *P. nigrispinus* nymphs after exposure to 33 and 50% neem oil suggests susceptibility of this predator to neem compounds. Increase duration of the nymph stage may affect reproductive fitness, because females with shorter lifespan lay higher numbers of eggs^29^. Thus, studies on collateral effects of neem formulations are necessary to detect their potential impact on non-target insects.

The survival <25% of fourth and fifth instar *P. nigrispinus* nymphs after treatment with concentrations of neem oil, suggests a degree of tolerance. Concentrations of neem oil between 0.1 and 10% cause mortality in various hemipteran pests and predators such as *Bemisia argentifolii* Bellows & Perring (Aphididae)^21^, *Clavigralla scutellaris* Westwood (Coreidae)^19^, *Macrolophus caliginosus* Wagner (Miridae)^11^, *Myzus persicae* Sulzer (Aphididae)^8^, *Nezara viridula* Linnaeus (Pentatomidae)^13^, *Nilaparvata lugens* Stal (Delphacidae)^47^, and *Picromerus bidens* Linnaeus (Pentatomidae)^49^. The bioneem is recommended in Brazil to control phytophagous insects at concentrations of 0.5 and 1%. Therefore, this product may be used against pests in the presence of *P. nigrispinus* if used in concentrations lower than 25%.

Several malformations, possibly related to defective molting, were observed from 1 to 50% of neem oil concentrations. Azadirachtin was established as an insect growth regulator with a novel mode of action. The basis for its mode of action was known to involve the neurosecretory–neuroendocrine pathway and perhaps other sites including cell cycle^46^. Studies on the insect growth regulatory mode of azadirachtin action in *Calliphora vicina* Robineau-Desvoidy (Diptera: Calliphoridae), *Manduca sexta* Linnaeus (Lepidoptera: Sphingidae), and *Oncopeltus fasciatus* Dallas (Hemiptera: Lygaeidae), show that JH biosynthesis and catabolism were affected by azadirachtin improving the insect growth regulatory effects^50^,^51^,^52^. Azadirachtin induces supernumerary molts, lack of black pigment and malformations^53^,^54^. Malformations on the legs, thorax and wings of *P. nigrispinus* adults exposed to neem oil in the third instar were similar to those found in the coccinellids *Chilochorus bipustulatus* and *Phroscymnus anchorago*^55^ and the lacewing *M. caliginosus*^11^. Nymphs of the phytophagous stink bug *Nezara viridula* Linnaeus (Hemiptera: Pentatomidae) exposed to commercial neem oils have malformations in the antennae, ocelli, tarsi, odoriferous glands, scutellum, genitalia and mouth parts^11^,^56^,^57^,^58^.

Ideally, phytochemical insecticides should be toxic to pests with low or no impact on predators. In this study, topical applications of neem oil showed low acute toxicity for *P. nigrispinus* nymphs, especially at concentrations <25%. However, sublethal effects, such as increased mortality rates of fourth and fifth instars, longer instar duration and body malformations indicate that the use of neem oil associated with biological control using the predator *P. nigrispinus* should be previously evaluated for the use in IPM.

## Methods

### Maintenance of Insect Culture

Nymphs and adults *P. nigrispinus* were obtained from mass rearing of the Laboratório de Controle Biológico do Instituto de Biologia Aplicada à Agropecuária (BIOAGRO, Universidade Federal de Viçosa, Minas Gerais, Brazil). They were maintained at 25 ± 2 °C at 75 ± 5% relative humidity and 12-h photophase. The insects were kept in wooden cages (30 × 30 × 30 cm) coated with nylon and glass, and received *ad libitum Tenebrio molitor* (L.) pupae (Coleoptera: Tenebrionidae), *Eucalyptus grandis* (W. Hill ex. Maiden) leaves and water^27^.

### Neem oil

Vegetable Bioneem is an organic Brazilian product composed of oil obtained from cold extraction of neem seeds, without the addition of solvents or pesticides, and certified by Ecocert Brazil as a natural insecticide with repellent properties. This product was developed with azadirachtin (25 g L^−1^) and others neem oil isomers concentrations for tropical regions. Ecocert Brazil is accredited by the Ministry of Agriculture, Livestock and Supply of Brazil and by international organizations according to ISO Guide 34 59. This provides Brazilian producers with licenses, and ensures unrestricted access to major world organic product markets.

### Comparative toxicity of neem oil and insecticides

Neem oil with 1800 to 2200 ppm of azadirachtin (Bioneem, Tecnologia Consultoria Indústria Comércio, Brazil) was compared with two different insecticides used as positive control in this study. The following commercial insecticides were tested at their maximum label rates: pyriproxifen (Tiger EC, Sumitomo Chemical Corporation, Brazil), 100 g L^−1^ and imidacloprid (Evidence WG, Bayer, Germany), 700 g L^−1^. These insecticides were diluted in 1 L water to produce a stock solution by adjusting 100 g L^−1^ per insecticide and to obtain the required concentrations. Insecticide efficacy was determined by calculating the lethal concentrations (LC_50_ and LC_90_) values under laboratory conditions for each formulation. Six concentrations of vegetable bioneem, pyriproxifen, imidacloprid besides the control (distilled water) were adjusted in 1 mL stock solution (treatments and distilled water): 1.56, 3.12, 6.25, 12.5, 25, and 50%. For each treatment, aliquots were taken from the stock solution and mixed with distilled water in 5 mL glass vials. Different concentrations of the treatments were applied in 1 μL of topical solution in the body of each individual of *P. nigrispinus*. Fifty third instar nymphs and fifty adults were used per concentration and were placed individually in glass vials (2 × 10 cm) with a cotton lid and maintained in the dark. The number of dead insects in each vial was counted after neem and insecticides exposure at intervals of 6 h over 6 days.

### Mortality test

Six concentrations of neem oil besides the control (liquid glycerin), were adjusted in 10 μL stock solution (neem oil and liquid glycerin): 0.5, 1, 20, 25, 33 and 50% aliquots were taken from the stock solution and mixed with liquid glycerine in 5 mL glass vials. Different neem oil concentrations were topically applied in 1 μL solution onto insect scutellum with an analytical 10 μL syringe. The glycerin was chosen due to applied solution adherence in the *P. nigrispinus* scutellum in order to ensure the absorption of the pesticide. Fifty-two individuals of third, fourth, fifth instar nymphs and adults (1:1 males and females proportion) of *P. nigrispinus* were used per concentration and individually placed in plastic containers (15 × 10 cm) with a perforated lid, fed on *T. molitor* pupae, *E. grandis* leaves, and water under laboratory conditions. First and second instar nymphs were not tested because they are too small to the volume of insecticide applied. The number of dead insects per concentration was daily counted after neem oil exposure until adult emergence.

## Development

The development of *P. nigrispinus* nymphs and adults was daily registered. To monitor nymph and adult development, 1,200 third-instar nymphs were placed individually in Petri dishes (90 × 15 mm) with moistened cotton ball and fed on *T. molitor* pupae. Nymphs were maintained at 25 ± 2 °C at 75 ± 5% RH and 12-h photophase until, fourth and fifth instars and adult emergence. Survival and duration of fourth and fifth instar nymphs, and adults, after emergence, were recorded. Longevity and survival were determined from third instar nymph with the six neem oil concentrations.

### Malformations

The number of malformed nymphs and adults was quantified per concentration after topical application of the neem oil on third instar nymphs. Severe morphological abnormalities of the *P. nigrispinus* adults were photographed.

### Statistics

The LC_50_ and LC_90_, and their confidence limits were determined by logistic regression based on the concentration probit-mortality^60^, with the program XLSTAT-PRO v.7.5 for Windows^61^. Mortality, development time and survival data of nymph and adult were analyzed by one-way ANOVA. Mortality variables were summarized in percentages and the data transformed to arcsine square root. Tukey’s Honestly Significant Difference test (HSD) was used for comparing the means at the 5% significance level (PROC ANOVA) using SAS v9.0^62^. The malformations of fifth instar nymphs and adults were compared by frequency of responses with chi-square test using SPSS v17.0 for Windows^63^.

## Additional Information

**How to cite this article**: Mourão, S. A. *et al*. Toxic effects of the neem oil (*Azadirachta indica*) formulation on the stink bug predator, *Podisus nigrispinus* (Heteroptera: Pentatomidae). *Sci. Rep.* **6**, 30261; doi: 10.1038/srep30261 (2016).

## Figures and Tables

**Table 1:** Lethal concentrations of the neem oil extract compared with pryproxifen (growth inhibitor) and imidacloprid (neurotoxic insecticide) on *Podisus nigrispinus* (Heteroptera: Pentatomidae) nymphs and adults.

Compounds	Stage	aLC	bEV	cCI	d*X*^*2*^
Neem oil	Nymph	50	14.98 (0.249*)	12.89 (0.214*)–17.43 (0.290*)	14.29
		90	29.14 (0.485*)	25.44 (0.423*)–34.61 (0.576*)	
	Adult	50	41.92 (0.698*)	35.31 (58.84*)–49.75 (0.829*)	27.96
		90	95.81 (1.513*)	82.84 (1.380*)–114.7 (1.911*)	
Pyriproxifen	Nymph	50	5.699	4.269–7.167	28.55
		90	16.85	14.24–20.95	
	Adult	50	7.345	5.589–9.189	28.51
		90	21.66	18.32–26.90	
Imidacloprid	Nymph	50	7.822	6.408–9.404	23.67
		90	18.91	16.27–22.86	
	Adult	50	17.03	13.88–20.72	25.64
		90	44.53	37.90–54.68	

Doses of compounds were topically applied.

^a^LC_50_ and LC_90_ concentrations causing 50 and 90% mortality; ^b^EV, Estimated value (mg mL^−1^; ^c^CI, Confidence interval (mg L^−1^); ^d^*X*^*2*^, Chi-square value for lethal concentrations and fiducial limits based on a log scale with significance level at *P* < 0.0001. *Estimated value (mg L^−1^) in ppm.

**Table 2:** Development (days) and survival (%) (mean ± SE) of fourth and fifth instar nymphs and adults *Podisus nigrispinus* (Heteroptera: Pentatomidae) submitted to topical application of different concentrations (%) of neem oil (25 ± 4 °C, 70 ± 5% RH and 12 h photophase).

**Neem oil concentrations (%)**	**Fourth instar**	**Fifth instar**	**Adult**
	Development (days)*		
Control	3.48 ± 0.1b	5.00 ± 0.2a	16.2 ± 0.5a
0.5	3.74 ± 0.2a	5.94 ± 0.3b	16.5 ± 0.1a
1	3.42 ± 0.2b	5.11 ± 0.2c	16.3 ± 0.1a
20	3.70 ± 0.2a	4.84 ± 0.2d	16.3 ± 0.3a
25	3.23 ± 0.3c	4.62 ± 0.2d	16.1 ± 0.4a
33	3.05 ± 0.2d	4.62 ± 0.2d	15.6 ± 0.3b
50	3.05 ± 0.2d	4.07 ± 0.2e	15.7 ± 0.7b
F_1,137_	2.19	5.10	1.45
P	0.0031	0.0001	0.0167
	Survival (%)*		
Control	94.2 ± 0.5a	98.8 ± 0.1a	99.7 ± 0.3a
0.5	93.4 ± 0.7a	99.4 ± 0.5a	96.7 ± 0.7a
1	94.9 ± 0.1a	95.8 ± 0.4a	96.1 ± 0.5a
20	83.5 ± 0.2b	84.8 ± 0.1b	96.3 ± 0.6a
25	72.3 ± 0.3c	76.4 ± 0.2c	91.1 ± 0.1b
33	60.3 ± 0.2d	64.62 ± 0.5d	85.6 ± 0.3b
50	55.8 ± 0.2e	54.3 ± 0.7e	85.8 ± 0.3b
F_1,137_	5.24	7.53	3.47
P	0.0001	0.0001	0.0019

^*^Means followed by the same letter within each column are not different according to the Tukey’s test (P < 0.05).

**Table 3:** Malformation (%) in fifth instar nymphs and adults *Podisus nigrispinus* (Heteroptera: Pentatomidae) after topical application of the neem oil extracts in the third instar (25 ± 4 °C, 70 ± 5% RH and 12 h photophase).

**Stage**	**Concentration (%)**	**n**	**Normal**	**Malformation**	***X***^***2***^
Nymph	Control	50	100	0	7.407
	0.5	40	98.1	1.9	
	1	37	94.7	5.3	
	20	35	92.3	7.7	
	25	31	88.4	11.6	
	33	27	80.7	19.3	
	50	26	78.4	21.6	
Adult	Control	50	100	0	50.52
	0.5	40	97.5	2.5	
	1	37	97.2	2.8	
	20	35	82.8	17.2	
	25	31	77.4	32.6	
	33	27	74.0	26.0	
	50	26	69.2	30.8	
